# The effectiveness of implementing the *Guideline for the Prevention of Mental Ill-health Problems at the Workplace* on health-outcomes, organizational and social risk factors: a cluster-randomized controlled trial in Swedish schools

**DOI:** 10.5271/sjweh.4108

**Published:** 2023-09-01

**Authors:** Anna Toropova, Andreas Rödlund, Christina Björklund, Liselotte Schäfer Elinder, Irene Jensen, Lydia Kwak

**Affiliations:** 1 Unit of Intervention and Implementation Research for worker health, Institute for Environmental Medicine, Karolinska Institute, Sweden.; 2 Department of Global Public Health, Karolinska Institute, Stockholm, Sweden.; 3 Centre for Epidemiology and Community Medicine, Region Stockholm, Stockholm, Sweden.; # These authors have contributed equally to this work and should be given equal credit as first authors.

**Keywords:** guideline adherence, implementation, mental health, occupational health, occupational safety, Sweden

## Abstract

**Objectives:**

This study aimed to compare the effectiveness of the multifaceted implementation strategy (multifaceted group) versus a discrete implementation strategy (discrete group) for implementing the Swedish *Guideline for the Prevention of Mental Ill-health Problems at the Workplace* on the primary intervention outcome – exhaustion – and secondary outcomes of stress, health, recovery, psychosocial safety climate, and social and organizational risk factors. Another aim was to examine whether the primary and secondary outcomes differed on the basis of guideline adherence levels, irrespective of the group.

**Methods:**

A cluster-randomized waiting-list controlled trial with 6- and 12-months follow-up was conducted among 19 Swedish public schools. Primary and secondary outcomes as well as guideline adherence were assessed by self-reported questionnaire. Linear mixed modeling was used to compare differences in outcomes between the groups from baseline to 6 and 12 months, and in relation to different adherence levels.

**Results:**

The trial comprised 698 employees (83.1%) participated. There were no differences between groups in the primary and secondary outcomes at 6 months, while at 12 months differences were observed for some outcomes to the advantage of the discrete group. Better guideline adherence was associated with improvements in exhaustion at 12 months and the secondary outcomes of psychosocial safety climate, work organization and job content, interpersonal relations and leadership, and recovery over 6 and 12 months.

**Conclusion:**

The multifaceted implementation strategy was no more effective than the discrete strategy in improving health outcomes or organizational and social work environment. However, higher adherence to the guideline was associated with larger improvements in health outcomes and organizational and social work environment, irrespective of the implementation strategy used.

Mental health problems (MHP) such as anxiety, depression, and stress-related disorders are common in the working population, resulting in individual suffering and high costs for employers and society (1, 2). There is a growing recognition that work-related MHP can be prevented by adequate management of organizational and social risk factors at the workplace (2–5). Evidence shows that these risks are best managed through a systematic and structured approach at the organizational level to identify and intervene on identified risk factors (6–8). Several global organizations, such as the Organization for Economic Cooperation and Development (9) and the World Health Organization (10), have adopted this notion and called for guidelines that can support employers with the systematic prevention of work-related MHP.

A recent systematic review (11) identified eight occupational health guidelines that include recommendations for an organizational approach toward preventing MHP at the workplace. Even though the recommendations of these guidelines vary to some extent, they follow a similar structure. In short, the employer should undertake a risk management procedure by (a) developing mental health policies including a systematic strategy, (b) ensuring cross-level commitment and involving key persons in the development of action plans, (c) having systematic structures for monitoring risks at the workplace, (d) promoting positive factors and minimizing risks, and (e) continuously evaluating and adapting their action plans (11). Even though guidelines are an essential part of preventing work-related MHP, research has shown that solely disseminating guidelines does not result in full implementation in practice (12) as multiple contextual determinants affect how efficiently guidelines are implemented. In order to facilitate adherence, implementation strategies that target these preidentified contextual determinants are needed. Knowledge regarding evidence-based strategies is currently lacking (13, 14). As a consequence, evidence-based practice is often not applied when aiming to prevent work-related MHP, which can negatively influence employees’ health. A highly ranked guideline in the review is the Swedish *Guideline for the Prevention of Mental Ill-health at the Workplace* (15). This evidence-based guideline has been developed in collaboration with employers, occupational health practitioners and researchers and complements the national provisions of the Swedish Work Environment Authority, which state that all employers are responsible for preventing mental ill-health and promoting a healthy work environment (15, 16).

Although guidelines are based on scientific evidence, the systematic review by Nexø et al (11) reported that few guidelines for the prevention of MHP at the workplace have been tested in practice for their effectiveness on improving organizational and social risk factors and health-related outcomes. Studies have evaluated the effectiveness of adherence to occupational health guidelines on the treatment of work-related MHP and have concluded that adherence to occupational health guidelines predicted earlier return to work from being sick-listed due to MHP (17, 18). However, to our knowledge, there are no studies that have tested the effect of an occupational health guideline on organizational and social risk factors for preventing MHP at the workplace. Thus, it is important to evaluate whether adherence to occupational health guidelines to prevent MHP results in an improved work environment and improved employee health (19, 20). Besides ensuring quality (19, 21), knowledge generated about the guideline’s effectiveness can motivate employers to work systematically to prevent work-related MHP.

In 2017, we conducted a cluster randomized controlled trial within a school setting to test the effectiveness of different implementation strategies on adherence to the recommendations of the *Guideline for the Prevention of Mental Ill-health at the Workplace* (22). Schools were selected due to the high prevalence of MHP among school personnel (23–25). The school’s work environment is characterized by high workload and demands, lack of recognition and support, and work-related conflicts, which are all known risk-factors for work-related MHP (26–28). Moreover, an inspection by the Swedish Work Environment Authority showed that many schools in Sweden have severe shortages in their occupational safety and health management (29). The trial showed no significant differences in adherence to the guidelines between those schools that received a multifaceted implementation strategy (further referred to as the multifaceted group) and those receiving a discrete strategy (further referred to as the discrete group), except for the adherence to one of the items of Recommendation 3 to the advantage of the discrete group (30).

The current study addresses the following research questions: (i) Is there a difference in the primary outcome of exhaustion and the secondary outcomes of health, stress, recovery, psychosocial safety climate, and organizational and social work-environment risk factors between the multifaceted and discrete implementation strategy at 6 and 12 months? (ii) Do the levels of exhaustion, stress, health, recovery, psychosocial safety climate, and social and organizational risk factors differ on the basis of the levels of guideline adherence at 6 and 12 months, irrespective of group? The second research question was formulated post-hoc as it was deemed important to examine whether adhering to a guideline that supports an organizational approach is associated with improvements in the organizational and social work environment in accordance with research evidence.

## Methods

### Study design and study population

This study reports the intervention outcome effectiveness of a 12-month cluster-randomized waiting-list controlled trial in Swedish public schools (comprising elementary, middle and high school levels). In Sweden, education is compulsory for 10 years starting at the age of 6 and continuing until grade 9 (15–16 years). The participating schools were randomized based on municipality and school size stratification into either multifaceted group or discrete group in a 1:1 ratio. The multifaceted strategy included an educational meeting, local implementation teams, workshops, and an iterative and evaluative strategy. The discrete strategy only included the educational meeting. After 12 months, the schools in the discrete group received the remaining strategies. The trial was registered on ClinicalTrials.gov (NCT03322839), assigned date: August 2017) and approved by the Ethical Committee of Stockholm (2017/984-31/5) (22).

The initial recruitment was at municipality level and followed a two-step process. First, advertisements were disseminated through newsletters, such as the Swedish Association of School Principals and Directors of Education and the Swedish Union of Teachers. Two municipalities showed an interest and agreed to participate. In the second step, the research team presented the study to principals in these two municipalities. One municipality had seven public schools, whereas the other had thirteen. The Swedish school system is tax-financed, and the municipality allocates resources to the schools (SFS:2010:800).

Several strategies were used to recruit participants at the school level. The research team visited each school to introduce the study. During this visit, the school personnel also had the opportunity to ask questions regarding the study. Those individuals absent at the presentation received a link to a recorded presentation. All participants received an information letter, including the study’s purpose, the research approach, voluntary participation, and information about the data collection process. Furthermore, individuals who agreed to participate received an informed consent form, which was completed and returned to the research team. All personnel employed by the school management were eligible for participation. A total of 698 individuals agreed to participate and answered the baseline questionnaire, which gives a response rate of 83.1%. As the guideline recommendations targeted the organizational level, an open cohort was employed in the current study, which means that participants were able to join the study at 6 and 12 months.

### Intervention components and implementation strategies

The implementation object was the *Guideline for the Prevention of Mental Ill-health at the Workplace* (15). This guideline offers a structured working model for managing organizational and social risks through three overarching recommendations: (i) workplaces should have well-established policies regarding organizational and social risk management, (ii) employers are aware of the link between organizational and social risks and mental ill-health, and (iii) workplaces continuously assess their organizational and social work environment and intervene on identified risks. The multifaceted implementation strategy consisted of four strategies. These were an educational day, local implementation teams, ongoing workshops, and an iterative and evaluative strategy. The discrete strategy included the educational day. Kwak et al (22) further describe the development of these implementation strategies.

*Educational day.* The education day was a full-day meeting (6.5 hours) carried out in October 2017 with all participating schools in each municipality. The educational meeting was held by one researcher with expertise in implementation research and one psychologist with expertise in occupational health. The education meeting included lectures, such as a presentation of the guideline, an introduction of barriers and facilitators to implementing the guideline, and a segment aimed at generating motivation through a set of motivational questions. The day also consisted of practical exercises and discussions to reflect upon implementing the guideline.

*Local implementation teams.* After the educational meeting, each school was instructed to form a local implementation team with those 3–5 individuals that participated in the educational day. These individuals were responsible for implementing the guideline at the school. The implementation team was intended to include members such as the principal, assistant principal, health and safety officers, and representatives from the personnel.

*Workshops.* In order to support the implementation teams, a series of five workshops (2.5 hours per workshop) were conducted. These workshops were intended to give the implementation teams knowledge and skills to implement the guideline. Each workshop focused on a specific guideline recommendation, where the implementation team received lectures and performed practical exercises related to the implementation of the recommendation. In addition, the schools were able to discuss their work with other schools.

*Plan-Do-Study-Act cycle.* The iterative and evaluative strategy employed in this trial was the Plan-Do-Study-Act (PDSA) strategy (31). At the first workshop, each implementation team started its first PDSA cycle by creating a detailed plan to implement the recommendation (plan) in between the workshops (do). At the next workshop, the implementation team studies how the plan worked out (study), adapts the plan if needed (act), and starts a new cycle with a new action plan or continues working with the revised plan.

### Outcome measures

The primary intervention outcome in the study was exhaustion, assessed with the four negatively phrased items from the Oldenburg Burnout Inventory Scale (32). Exhaustion is one of the most common diagnoses within MHP in Sweden (33), and therefore chosen as the primary intervention outcome. Secondary intervention outcomes included the following health-related outcomes: self-perceived health (34), self-reported stress (35) and recovery (‘Do you feel you have recovered and thoroughly rested when you start work after a vacation/holiday?) (36). Moreover, the schools’ organizational and social work environment was assessed by demands at work, work organization and job content, interpersonal relations and leadership, and work-life conflict [all four constructs stemming from the validated Copenhagen Psychosocial Questionnaire (37)]; work performance impairment (38) and work engagement (39). Finally, psychosocial safety climate was measured by the Psychosocial Safety Climate scale (40). All outcomes were measured at baseline, 6, and 12 months. A more detailed description of the measures, including answering categories and direction of the scales is provided in table 2 and the study protocol (22).

**Table 2:** Primary and secondary intervention outcome measures at baseline measurement, and 6-months- and 12-months follow-up and absolute changes between baseline and follow-ups

Outcome	Multifaceted group, M (SD)								Discrete group, M (SD)						
	T0 (N=334)		T1 (N=297)		T2 (N=323)		Absolute change		T0 (N=364)		T1 (N=327)		T2 (N=353)		Absolute change
	Mean (SD)		Mean (SD)		Mean (SD)		6/12 months		Mean (SD)		Mean (SD)		Mean (SD)		6/12 months
Exhaustion (1–5) ^a^	2.76 (0.69)		2.70 (0.70)		2.75 (0.71)		-0.06/-0.01		2.70 (0.74)		2.60 (0.73)		2.59 (0.73)		-0.10/-0.11
Self-rated general health (1–5) ^b^	3.28 (0.96)		3.33 (0.99)		3.27 (0.99)		0.05/-0.01		3.41 (1.02)		3.44 (1.03)		3.48 (1.02)		0.03/0.07
Perceived stress (1–5) ^a^	3.36 (1.16)		3.09 (1.19)		3.25 (1.19)		-0.27/-0.11		3.19 (1.19)		2.92 (1.17)		2.97 (1.22)		-0.27/-0.22
Psychosocial safety climate (12–60) ^b^	26.47 (9.10)		28.54 (9.84)		27.72 (9.95)		2.07/1.25		30.07 (9.25)		31.79 (9.83)		32.04 (8.95)		1.72/1.97
Demands at work (1–5) ^a^	3.59 (0.62)		3.42 (0.59)		3.53 (0.64)		-0.17/-0.06		3.49 (0.62)		3.37(0.60)		3.43 (0.59)		-0.12/-0.06
Work organization and job content (1–5) ^b^	3.31 (0.47)		3.29 (0.51)		3.32 (0.51)		-0.02/0.01		3.39 (0.49)		3.39 (0.51)		3.47 (0.48)		0.00/0.08
Interpersonal relations/ leadership (1–5) ^b^	3.31 (0.65)		3.30 (0.66)		3.20 (0.69)		-0.01/-0.11		3.48 (0.61)		3.47 (0.61)		3.39 (0.61)		-0.01/-0.09
Recovery (1–5) ^b^	3.47 (0.99)		3.60 (0.97)		3.43(1.00)		0.13/-0.04		3.45 (1.01)		3.62 (0.98)		3.69 (1.02)		0.17/0.24
Work performance impairment (1–10) ^a^	4.75 (2.19)		4.53 (2.23)		4.73 (2.19)		-0.22/-0.02		4.58 (2.35)		4.68 (2.41)		4.64 (2.32)		0.10/0.06
Work-life conflict (1–4) ^a^	2.35 (0.88)		2.24 (0.84)		2.32 (0.85)		-0.11/-0.03		2.33 (0.90)		2.18 (0.84)		2.22 (0.84)		-0.15/-0.11
Work engagement (1–7) ^b^	6.05 (0.83)		6.04 (0.78)		5.92 (0.91)		-0.01/-0.13		5.98 (0.94)		5.94 (0.95)		6.06 (0.94)		-0.04/0.08

^a^ Higher scores indicate a more negative condition. ^b^ Higher scores indicate a more positive condition

### Adherence index

A guideline adherence index was calculated based on participants’ responses to the adherence indicators to each of the guideline recommendations, which were dichotomized into ‘Adherence’ (those responding ‘strongly agree’ and ‘agree’ to guideline adherence indicators) and ‘Non-adherence’ (those responding ‘strongly disagree’, ‘disagree’, ‘neither agree nor disagree’ or ‘I don’t know’). Adherence was coded as 1, while non-adherence was coded as 0 (30). The adherence index was created by summing up participants’ adherence scores on each of the eight adherence indicators. The index values thus ranged from 0–8.

### Sample size and randomization

Power calculations were performed at the employee level adjusting for school-clustering. The sample size calculation was conducted prior to the study to detect potential improvements in the primary outcome with 30% among employees in the multifaceted group in contrast with the discrete group. Based on an alpha significance level of 0.05, desired power of ≥80%, and intraclass correlation coefficient of 0.005, approximately 400 individuals in total were required: 200 in the multifaceted group and 200 in the discrete group. It was calculated that this would require ≥18 schools.

In total, 334 participants were included in the multifaceted group clustered within 10 schools, whereas 364 participants were in the discrete group clustered within 9 schools. The randomization of schools was conducted through a computer-generated randomization-list before the baseline measure. An independent statistician not involved in the project performed the randomization. Neither school management nor school personnel were blinded.

### Statistical analysis

**Figure 1:**
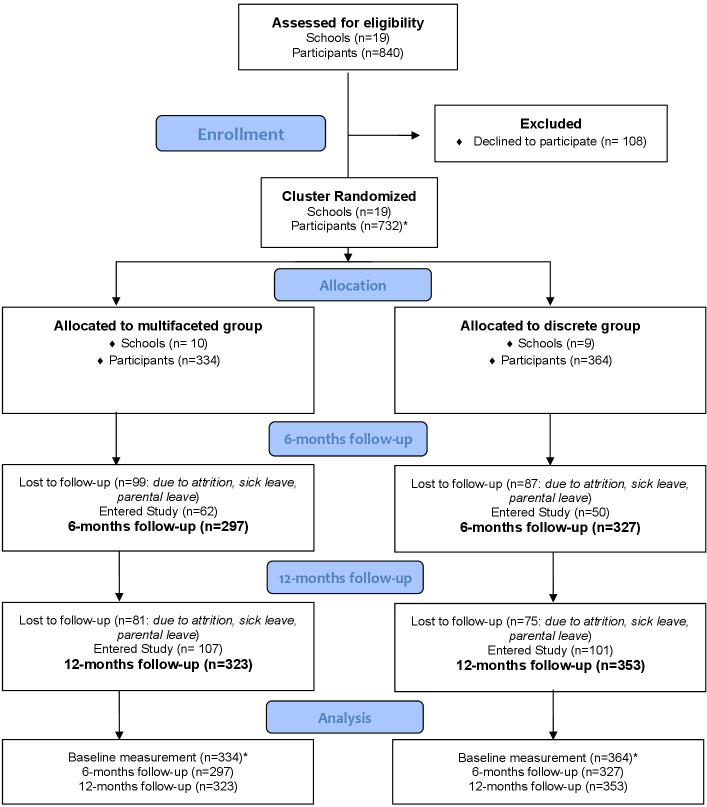
CONSORT 2010 flow diagram. *After enrollment, participants were excluded prior to the analysis due to participants only completing the demographic part of the questionnaire, moreover, some participants withdrew after completion of the questionnaire and were excluded from the study (N=34)

Participants’ demographic characteristics, as well as primary and secondary outcomes at baseline are presented using frequencies, means and standard deviations, or percentages. Further, absolute changes in primary and secondary outcomes between baseline and 6 and 12 months (within group) were calculated. As the study employed an open cohort, the number of participants differs at baseline, 6 and 12 months (for flowchart see figure 1).

To address the first research question, whether there was a difference in the primary intervention outcome exhaustion and the secondary intervention outcomes between the multifaceted and discrete groups from baseline to 6 and 12 months, intervention effects for primary and secondary outcomes were tested by means of linear mixed modeling. Group and time variables were introduced as fixed factors in the model, while a group×time interaction was an indicator of the intervention effect. Baseline values of the primary and secondary outcomes were controlled for in the model. The nested data structure was accounted for by using a person-specific random intercept to model the within-subject clustering over time. In addition, a model with a school-specific random intercept to account for clustering within schools was tested, however, it did not explain any additional variance beyond person-specific clustering over time and was therefore not used in further analysis.

To answer the second research question, an adherence index was added in the model as a covariate. In order to confirm that the results were not influenced by the way adherence index was constructed, we conducted an additional analysis with adherence index as a categorical variable with three categories: low adherences (values 0–2), moderate adherence (values 3–5), and high adherence (values 6–8). The considerably smaller sample size for the second research question is due to the fact that adherence indicators for guideline recommendation 3 were preceded by the filter question, resulting in a fewer number of participants responding to these items.

As schools are known for high levels of school personnel turnover – during the trial, two schools in the multifaceted group and one in the discrete group underwent organizational changes (entailing a change of the principal and a transfer of the upper-level of education to another school) – it was hypothesized that participants’ work experience at the school and school organizational change could account for differences in the primary and secondary outcomes. Work experience at the school and organizational change were therefore included in the model as fixed covariates. Separate models were fitted for each of the outcomes.

Estimates of intervention effects were reported as regression coefficients with 95% confidence intervals (CI), with alpha significant levels set to 0.05 for two-sided statistical tests. Data were analyzed with the help of IBM SPSS Statistics 27, (IBM Corp, Armonk, NY, USA).

## Results

### Descriptive statistics

Descriptive statistics of the study participants’ characteristics at baseline are presented in table 1.

**Table 1:** Participant characteristics at baseline. [SD=sustainable development].

School personnel characteristics		Multifaceted group (N=336)				Discrete group (N=362)		
		N	%	Mean (SD)		N	%	Mean (SD)
Age		325		47.26 (11.95)		357		44.84 (11.68)
Gender (female)		254	76.5			272	74.9	
Professional title								
	Teacher	232	76.3			248	72.3	
	Other school personnel	72	23.7			95	27.7	
Education level								
	Basic education	9	2.7			10	2.8	
	Secondary education	59	17.7			64	17.8	
	University education	256	77.3			272	75.8	
	Post-graduate education	7	2.1			13	3.6	
Work experience in the field (years)								
	<5	85	25.7			108	29.8	
	5–14	94	28.4			115	31.8	
	15–24	72	21.6			78	21.5	
	25–34	46	13.8			36	9.9	
	≥35	34	10.2			25	6.9	
Work experience in current school (years)								
	<5	196	60.9			226	65.5	
	5–14	62	19.3			84	24.3	
	15–24	45	13.9			27	7.8	
	25–34	14	4.3			7	2.0	
	≥35	5	1.5			1	0.3	

### Primary and secondary outcomes at baseline and across follow-ups

Primary and secondary outcome measures at baseline, and 6 and 12 months are shown in table 2. Moreover, the table includes absolute changes between baseline and follow-up.

Improvements are observed in both multifaceted and discrete groups regarding the primary and the majority of secondary outcomes at six months, with larger absolute improvements for self-reported health, work demands, psychosocial safety climate, and work performance impairment in favor of the multifaceted group. At 12 months, greater absolute improvements are seen for all of the outcomes, except for work performance impairment, in favor of the discrete group.

The comparative effectiveness of the multifaceted implementation strategy versus the discrete strategy on the primary and secondary intervention outcomes is presented in table 3.

**Table 3:** The comparative effectiveness between the multifaceted and discrete group on the primary and secondary intervention outcomes (N=667). [B=unstandardized coefficient; CI=confidence intervals]. **Bold indicates statistical significance.**

Outcome	Intervention effect 6 months ^a^	P-value	Intervention effect 12 months ^a^	P-value
	B (95% CI)		B (95% CI)	
Exhaustion	0.02 (-0.06−0.11)	0.543	**0.10 (0.01−0.20)**	**0.038**
Self-rated general health	0.10 (-0.03−0.22)	0.139	-0.09 (-0.23−0.04)	0.180
Perceived stress	-0.04 (-0.21−0.13)	0.640	**0.23 (0.05−0.42)**	**0.014**
Psychosocial safety climate	0.82 (-0.53−2.17)	0.233	-0.42 (-1.8−0.98)	0.557
Work demands	-0.02 (-0.09−0.04)	0.482	0.04 (-0.03−0.12)	0.275
Work organization and job content	0.03 (-0.03−0.09)	0.320	0.004 (-0.06−0.07)	0.886
Interpersonal relations/ leadership	0.003 (-0.08−0.08)	0.947	-0.05 (-0.13−0.04)	0.293
Recovery	-0.02 (-0.17−0.12)	0.734	**-0.31 (-0.46−0.17)**	**0.001**
Work performance impairment	0.10 (-0.44−0.46)	0.967	0.07 (-0.39−0.53)	0.762
Work-life conflict	0.04 (-0.06−0.14)	0.421	**0.11 (0.003−0.21)**	**0.044**
Work engagement	0.04 (-0.07−0.16)	0.458	**-0.17 (-0.29−0.04)**	**0.008**

^a^ Linear mixed models; multifaceted group versus discrete group mean difference, adjusted for work experience at the school and school organizational change

At 6 months, no statistically significant differences were observed in either primary or secondary outcomes (adjusted mean differences) between the multifaceted and discrete group. At 12 months, a statistically significant difference was observed for exhaustion, meaning that exhaustion was higher in the multifaceted group than in the discrete group. For the secondary outcomes, a statistically significant difference was found in perceived stress, with higher levels of perceived stress observed in the multifaceted group at 12 months. Moreover, statistically significant differences were also observed in recovery, work-life conflict and work engagement to the advantage of the discrete group.

### Association of guideline adherence with the primary and secondary outcomes

*Guideline adherence – continuous variable.* The association of the levels of guideline adherence as a continuous variable, irrespective of group, with the primary and secondary intervention outcomes at 6 and 12 months respectively are presented in table 4.

**Table 4:** The association of guideline adherence as a continuous variable with the primary and secondary outcomes (N=241). [B=unstandardized coefficient; CI=confidence interval]. **Bold indicates statistical significance.**

Outcome	Adherence at 6 months ^a^	P-value	Adherence at 12 months ^a^	P-value
	B (95% CI)		B (95% CI)	
Exhaustion	-0.01 (-0.02−0.01)	0.269	**-0.02 (-0.03−0.00)**	**0.045**
Self-rated general health	0.02 (-0.004−0.13)	0.103	**0.03 (0.001−0.05)**	**0.038**
Perceived stress	**-0.04 (-0.06−0.009)**	**0.009**	-0.02 (-0.05−0.01)	0.285
Psychosocial safety climate	**0.62 (0.42−0.83)**	**<0.001**	**0.60 (0.38−0.82)**	**<0.001**
Work demands	-0.01 (-0.02−0.003)	0.138	-0.01 (-0.02−0.01)	0.402
Work organization and job content	**0.02 (0.01−0.03)**	**<0.001**	**0.02 (0.01−0.03)**	**0.002**
Interpersonal relations/ leadership	**0.03 (0.02−0.04)**	**<0.001**	**0.02 (0.01−0.04)**	**0.002**
Recovery	**0.02 (0.00−0.05)**	**0.047**	**0.03 (0.001−0.05)**	**0.040**
Work performance impairment	-0.05 (-0.13−0.03)	0.198	-0.08 (-0.16−0.003)	0.058
Work-life conflict	-0.01 (-0.03−0.004)	0.156	-0.02 (-0.04−0.001)	0.065
Work engagement	**0.02 (0.004−0.04)**	**0.018**	0.01 (-0.01−0.03)	0.541

^a^ Linear mixed models; adjusted for work experience at the school and school organizational change

A positive association was found between guideline adherence and the primary outcome exhaustion at 12 months, ie, one point increase in guideline adherence is related to 0.02 index points reduction in exhaustion. With regards to secondary outcomes, a positive association between guideline adherence and psychosocial safety climate is present at both 6 and 12 months, with one point increase in guideline adherence associated with better psychosocial safety climate. A similar pattern is seen for work organization and job content, interpersonal relations and leadership as well as recovery at both 6 and 12 months, with higher levels of guideline adherence related to improvements in the above outcomes. Finally, a positive association of guideline adherence can be observed with perceived stress (higher levels of guideline adherence related to lower stress levels) and work engagement (higher levels of guideline adherence associated with higher levels of engagement at 6 months), as well as for self-reported health (higher levels of guideline adherence related to better in health) at 12 months.

*Guideline adherence – categorical variable.* The results regarding the association of guideline adherence as a categorical variable with the primary and secondary intervention outcomes are presented in table 5.

**Table 5:** The association of guideline adherence as a categorical measure and the primary and secondary intervention outcomes (N=241) [B=unstandardized coefficient; CI=confidence intervals]. Moderate and high adherence are presented in relation to low adherence. **Bold indicates statistical significance.**

Outcome (guideline adherence)		Adherence at 6 months ^a^	P-value	Adherence at 12 months ^a^	P-value
		B (95% CI)		B (95% CI)	
Exhaustion					
	High	-0.06 (-0.17–0.06)	0.350	**-0.15 (-0.28–0.03)**	**0.015**
	Moderate	-0.003 (-0.09–0.08)	0.945	-0.01 (-0.09–0.07)	0.783
Self-rated general health					
	High	0.16 (-0.01–0.33)	0.058	**0.22 (0.03–0.40)**	**0.023**
	Moderate	0.04 (-0.08–0.17)	0.484	0.03 (-0.09–0.16)	0.593
Perceived stress					
	High	-0.19 (-0.41–0.03)	0.092	-0.17 (-0.41–0.07)	0.165
	Moderate	-0.14 (-0.30–0.02)	0.091	0.01 (-0.15–0.18)	0.873
Psychosocial safety climate					
	High	3.73 (2.09–5.38)	<0.001	**3.96 (2.23–5.69)**	**<0.001**
	Moderate	2.14 (0.94–3.35)	<0.001	**1.51 (0.35–2.66)**	**<0.001**
Work demands					
	High	-0.06 (-0.14–0.03)	0.175	-0.02 (-0.13–0.08)	0.661
	Moderate	-0.02 (-0.08–0.04)	0.573	-0.04 (-0.11–0.03)	0.258
Work organization and job content					
	High	**0.13 (0.05–0.20)**	**0.001**	**0.10 (0.02–0.18)**	**0.016**
	Moderate	0.05 (-0.01–0.11)	0.088	0.04 (-0.01–0.10)	0.128
Interpersonal relations leadership					
	High	**0.20 (0.11–0.30)**	**<0.001**	**0.16 (0.04–0.27)**	**0.009**
	Moderate	**0.08 (0.01–0.15)**	**0.022**	**0.09 (0.01–0.17)**	**0.026**
Recovery					
	High	0.16 (-0.03–0.35)	0.090	**0.21 (0.03–0.40)**	**0.026**
	Moderate	0.10 (-0.04–0.24)	0.168	0.02 (-0.11–0.15)	0.790
Work performance impairment					
	High	-0.12 (-0.79–0.54)	0.716	-0.49 (-1.10–0.11)	0.108
	Moderate	-0.39 (-0.81–0.03)	0.067	**-0.46 (-0.89–0.04)**	**0.034**
Work-life conflict					
	High	-0.06 (-0.18–0.06)	0.344	**-0.19 (-0.33–0.05)**	**0.008**
	Moderate	-0.02 (-0.11–0.07)	0.700	-0.03 (-0.13–0.06)	0.476
Work engagement					
	High	**0.16 (0.03–0.30)**	**0.021**	0.01 (-0.15–0.17)	0.900
	Moderate	0.01(-0.09–0.12)	0.827	-0.02 (-0.12–0.09)	0.782

^a^ Linear mixed models; adjusted for work experience at the school and school organizational change

As for the primary outcome of exhaustion, participants in schools with high levels of guideline adherence (in contrast with low levels) reported lower exhaustion at 12 months. With regards to the secondary outcomes, better psychosocial safety climate and interpersonal relations and leadership were related to both moderate and high levels of adherence at 6 and 12 months. A consistent positive association of high levels of guideline adherence across measurement points, similar to that of psychosocial safety climate, was also present for work organization and job content. A less consistent, although favorable, association of high levels of guideline adherence was found for work engagement at 6 months and self-rated health, recovery, and work-life conflict at 12 months.

## Discussion

In the present study, we aimed to investigate whether there were differences in the primary and secondary outcomes between the multifaceted and discrete implementation strategy at 6 and 12 months. Results showed no differences for either primary or secondary outcomes at 6 months, while at 12 months differences between the groups were found for most of the outcomes, however unexpectedly to the advantage of the discrete group. Next, we examined whether the level of adherence was related to changes in the organizational and social work environment in schools, regardless of the group. Results showed that the level of adherence to the guideline was associated with improvements in several individual and organizational level outcomes at both 6 and 12 months, ie, higher levels of adherence were related to better outcomes.

When looking at the intervention effect at 12 months follow-up, improvements in the primary outcome exhaustion and secondary outcomes stress, recovery, work-life conflict and work engagement were to the advantage of those schools receiving the discrete implementation strategy. These findings are in line with the results on implementation outcomes as reported previously in Toropova et al (30). In that study of the same trial, contrary to our expectations, no significant differences in guideline adherence were found between schools that received a multifaceted implementation strategy and those receiving a discrete strategy, except for the adherence to one item of Recommendation 3 (workplaces should continuously assess their organizational and social work environment and intervene on identified risks) to the advantage of the discrete group at 12 months (30). The findings of the present study are consistent with the above results and are equally unexpected. It is challenging to explain why the discrete group showed better outcomes at 12 months. Research suggests that the multifaceted strategy targeting multiple implementation determinants would provide better support, therefore leading to better implementation compared to the discrete strategy (41). One of the potential reasons for the multifaceted strategy not demonstrating the anticipated effect could be the lack of a more structured implementation support to schools, which could be addressed by, for instance, engaging an internal facilitator at municipality level (30). Results of the present study do not allow to conclude which strategy is the best. Further studies based on a larger sample, which additionally test implementation mechanisms, are needed.

Our findings on the association of guideline adherence and health-related outcomes, psychosocial safety climate, and organizational and social work-environment factors confirm the general assumption in the field that intervention effectiveness is dependent on implementation effectiveness; without successful implementation, intervention effects are unlikely to occur (12, 42). Higher adherence was related to lower exhaustion and stress, while it was positively related to health, psychosocial safety climate, work organization and job content, interpersonal relations and leadership, recovery, and work engagement. Even though the study was not designed to test the effect of guideline adherence, the results give an indication that working according to the guideline is beneficial for multiple health-related outcomes as well as the organizational and social work environment.

Our findings can be compared to those few studies of organizational-level interventions that have been conducted within a school-setting. A 2015 Cochrane systematic review on the effectiveness of organizational-level interventions for improving well-being and reducing work-related stress among teachers showed low-quality evidence for improvements in teacher well-being (43). Only two of the four included studies evaluated the effectiveness of changing organizational characteristics, and no significant effects were found on burnout, job-related anxiety and job-related depression (43). A quasi-experimental controlled trial evaluated an organizational-level participatory action approach targeting risks in the school work environment, and observed no statistically significant effects of the intervention neither on the primary outcome, the need for recovery, nor on the secondary outcomes (44). A recent multiple-case study in five schools evaluating implementation success of an organizational-level intervention with similar components as included in the Guideline for the prevention of mental ill-health, reported a favorable effect of most intervention components on stress levels and job demands over time (42). Finally, a study evaluating the effectiveness of an organizational intervention among pre-school personnel, including implementation support through implementation teams, similar to our study, found no statistically significant improvements in exhaustion, sleep disturbances and job satisfaction (45). Results of our study show no statistically significant difference between groups on the primary outcome exhaustion nor on the secondary outcomes at 6 months yet demonstrate statistically significant differences to the advantage of the discrete group at 12 months, which is partly in line with the results of the study by Framke et al (45).

In line with the study by Bakhuys Roozeboom et al (42), our results demonstrate that adherence to guideline recommendations is essential for improvement in both organizational and health-related factors. Despite the relatively small effect sizes, participants in schools with higher levels of guideline adherence show improvements in the primary outcome exhaustion, as well as in the majority of the secondary outcomes: health, stress, psychosocial safety climate, work organization and job content, interpersonal relations and leadership, recovery and work engagement. A beneficial association of guideline adherence and outcomes at individual level was observed irrespective of whether continuous or categorical measure of guideline adherence was used.

### Strengths and limitations

The main strength of the study is the cluster randomized waiting-list control design evaluating the intervention effectiveness. This study fills the knowledge gaps on the effectiveness of implementation support of a guideline for the prevention of mental ill-health on health-related outcomes and social and organizational risk factors at the workplace, as well as the association of the guideline adherence with the above outcomes. The majority of the studies evaluating organizational-level interventions, as recommended in the implemented guideline, have been identified as having a high risk of bias making it difficult to draw conclusions on the causality of the interventions. Another strong feature of this study includes the high response rate of 83% of personnel within the participating schools. The participants are therefore likely to be a representative sample of these schools. Finally, a strength of the study is that implementation support was evaluated as recommended by Schelvis et al (44).

A limitation of the present study is the relatively short follow-up period of 12-months, as organizational changes often require a long time to take place. To be able to observe larger effects on risk factors in the school’s work environment as a result of these organizational changes, a timespan of more than 12 months is most likely needed (46). Another limitation is related to the fact that guideline adherence measures combined adherence scores on all of the recommendations. Risk factors within the social and organizational work environment affected by the intervention likely depend on the recommendation being implemented. Moreover, guideline adherence was measured based on participants’ individual perceptions and objective measures are warranted in future research.

### Concluding remarks

There were no differences between groups in the primary and secondary outcomes at 6 months, while at 12 months differences were observed for some outcomes to the advantage of the discrete group. However, a positive association of adherence to guideline recommendations was found at both 6 and 12 months for the majority of the intervention outcomes irrespective of the group. Better implementation, as manifested by adherence to guideline recommendations in this study, was clearly related to improvements in negative exhaustion and other health-related outcomes, psychosocial safety climate as well as social and organizational risk factors in the school work environments. These findings confirm that adhering to evidence-based guidelines for the prevention of work-related MHP can result in improvement in health and organizational and social risk factors in the work environment. Future studies should examine which implementation strategies can lead to better adherence to such guidelines.
